# Music for displaced dyads: a mixed methods feasibility study on the impact of music therapy on the mental health and wellbeing of Ukrainian refugee families

**DOI:** 10.3389/fpsyt.2025.1707023

**Published:** 2025-10-24

**Authors:** Letitia Slabu, Elizabeth Coombes, Anthony M. A. Mangiacotti, Tamar Hadar, Fabia Franco

**Affiliations:** ^1^ Department of Psychology, Middlesex University, London, United Kingdom; ^2^ Faculty of Life Sciences and Education, University of South Wales, Newport, United Kingdom; ^3^ Faculty of Social Welfare and Health Sciences, University of Haifa, Haifa, Israel

**Keywords:** music therapy, mental health, trauma, displacement, music group, respiratory sinus arrhythmia

## Abstract

**Background:**

Global displacement has reached unprecedented levels, with refugee mothers and children particularly vulnerable to psychological distress. Following the war in Ukraine, many families face trauma, disrupted parenting, and limited access to mental health services. Music therapy (MT) offers a non-pharmacological, culturally adaptable approach to support psychosocial wellbeing. This feasibility study explored the impact of a dyadic MT intervention on Ukrainian refugee caregivers and their children resettled in the UK.

**Methods:**

Four groups of 4–6 caregiver-child dyads participated in an 8-week improvisational MT program, co-designed with caregivers and culturally tailored. A mixed-methods approach included: (1) quantitative pre/post measures of PTSD, depression, anxiety, wellbeing, cognitive functioning, parenting self-efficacy, musical home environment, and social connectedness; (2) physiological assessment of respiratory sinus arrhythmia (RSA) as an index of autonomic regulation; and (3) post-intervention semi-structured interviews with caregivers.

**Results:**

Significant improvements were observed in caregivers’ PTSD, depression, anxiety, and cognitive functioning. RSA data indicated increased parasympathetic activity, suggesting improved emotional regulation. Non-significant trends emerged in parenting and home musical engagement. Qualitative analysis identified enhanced child communication, socio-emotional functioning, and transference of musical engagement into the home.

**Conclusion:**

This study is the first to demonstrate the feasibility and potential efficacy of MT for improving mental health and parent-child dynamics among displaced Ukrainian families. Findings support MT as a low-cost, trauma-informed, and scalable intervention. Further research is needed to evaluate its impact in larger, culturally diverse refugee populations through randomized controlled trials.

## 1 Introduction

In Europe, the war in Ukraine has created one of the most significant humanitarian crises since the World War II, forcing nearly six million Ukrainian people, mostly women and children, to seek refuge across the European Union (1). Out of these, 4.3 million are children, representing nearly half of Ukraine’s child population (2). This unprecedented displacement has far reaching consequences for family life, creating profound instability and uncertainty that affect safety, daily routines, emotional wellbeing, and overall sense of security. Families with small children are especially vulnerable as such experiences affect children’s key developmental stages, caregiver’s mental health, and the parent–child relationship (3–5).

Current evidence suggests MT can be effective in reducing trauma-related symptoms (6–9); however, few studies have examined group MT for displaced individuals (10–15), and none have focused specifically on its application within a family context involving caregiver–child dyads. Such interventions have the potential to support both generations simultaneously in displacement settings, while also contributing positively to the host society.

Displaced individuals endure various stressors: a) limited self-protection resources, exposure to violence, death, and other military and war trauma; b) profound personal, material and symbolic losses of family members and loved ones, security, homes, health, socioeconomic status, hobbies and memories as well as; c) challenges like cultural shock, poverty, uncertainty related to temporary protection status, racism and discrimination (16). Additional hardships arise from the disruption of social support networks, often resulting in deep feelings of loneliness and social isolation (17, 18). War-related stress itself often continues via exposure to the news, and worries about, or loss of family members who still reside in the conflict area. These factors can lead to profound emotional distress and mental health problems (19, 20).

### 1.1 Mental health impact of war trauma exposure and displacement

It is indeed well documented that there is a high rate of mental health conditions after migration experiences (3), particularly post-traumatic stress disorder (PTSD), anxiety, depression (21, 22) and psychoses (23). In fact, approximately one in three refugees pass the diagnostic threshold for PTSD, depression and anxiety (3, 24–26). This broader trend is further reflected in research on the Ukrainian population, where conflict-related displacement has not only contributed to new psychological distress but has also intensified existing mental health conditions (27, 28). The results indicated that 32% of individuals internally displaced by the hostilities since 2014 showed signs of PTSD, while 22% reported symptoms of depression, and 17% experienced anxiety (29, 30).

Despite parents’ efforts to protect them, children often share the same adverse experience with their parents. As primary caregivers, refugee parents play a crucial role in supporting their children’s health and care needs (31). Women and children together constitute roughly half of the world’s forcibly displaced population (1, 32). While most research and much frontline care has focused on mothers, a growing body of work shows that fathers’ mental health and caregiving behaviour also play a critical role in shaping family adjustment when fathers are present (33–35). In conflicts where adult men are required to stay and fight or are legally barred from leaving, many refugee households become female-headed, compounding mothers’ perinatal stress and increasing the risk of psychiatric problems (36, 37). Ukraine is a current example where martial-law, enacted on 24^th^ of February 2022, requires men aged 18 to 60 to remain in the country, resulting in majority of externally displaced households being led by women. Large-scale monitoring as well as empirical studies have evidenced a link between this enforced family separation and heightened psychological distress among mothers and children (38–40). Comparable patterns have been documented elsewhere. Father absence predicts poorer mental-health outcomes for Syrian refugee families (41) and for “left-behind” children in low- and middle-income countries (42). As a result, refugee mothers experience disproportionately high rates of depression, anxiety, post-traumatic stress, and suicidality (43–45). A recent meta-analysis reported a peri-natal depression prevalence of 33.5% (46) among migrant population, that is twice the rate of the non-migrant population.

Regardless of the various mental health challenges these mothers experience, they are less likely to seek professional support because of stigma, language and cultural barriers, inadequate and limited access to existing services (47). Moreover, they frequently prioritize their children’s needs over their own wellbeing, often at a personal cost (48). However, this selfless act may have unintended consequences, as caregiver trauma and mental health issues are known risk factors that affect children’s emotional and behavioural outcomes (49–51). Parental trauma can profoundly shape the entire family context by influencing the quality of family relationships, communication patterns, and parenting behaviours. Several meta-analyses have indicated that parental PTSD symptoms are linked to negative parenting behaviours, including overprotection and emotional detachment (52), as well as difficulties in emotional attunement that reduce sensitivity to children’s emotional needs and impair parent-child communication (53). Furthermore, Gupta et al. (54) in their systematic review, report that these disruptions can impair family functioning and contribute to increased conflict and reduced cohesion.

These patterns are also evident in displacement contexts, where parental trauma has a cascading effect on children’s mental health. For example, empirical findings among Syrian displaced families showed that poor parental mental health predicts children’s emotional and behavioural problems (55), while exposure to parental war trauma was associated with increased conduct problems and hyperactivity in children (31). A recent dyadic study with Ukrainian families displaced to Israel following the 2022 invasion reinforces this pattern, showing that (56) higher parental PTSD and distress were significantly associated with elevated child PTSD symptoms, general distress, and externalising behaviours. Children of parents with high PTSD symptoms displayed markedly greater difficulties than their peers, reinforcing findings from other conflict-affected settings.

The various strains displaced families endure can disrupt parenting practices and compromise the emotional environment necessary for healthy child development, increasing the risk of emotional and mental health issues for these children (49, 50). Childhood mental health problems can have long-lasting negative outcomes for individuals, including both physical and psychological health issues, suicidality and substance use across the lifespan as well as increased risks of delinquency and unemployment (57–61). Scientific advances in neurobiology have shown that complex trauma, particularly during developmentally vulnerable stages (62), can have lasting effects on the brain, mind, and body thereby shaping one’s capacity for resilience (63, 64). These outcomes have far-reaching implications for children’s development, as well as for family dynamics, community wellbeing and society at large.

It is therefore crucial to develop early interventions that simultaneously address the psychosocial wellbeing of displaced parents and their children. Thus, the present study focuses on family dyads (caregiver-child) who are displaced from Ukraine and who are newly resettled in the United Kingdom aiming to support their mental health and increase their wellbeing in a music therapeutic framework. MT offers a non-pharmacological approach that could be particularly well-suited for this purpose.

### 1.2 Music therapy for displaced people

There is growing evidence that MT can effectively support mental health, improve socio-emotional functioning, and foster a sense of community, even across cultural and linguistic boundaries (65, 66). In response to the adverse experiences associated with living in and escaping conflict zones, much of the published literature on music and MT focuses on alleviating symptoms of trauma and depression among displaced populations (10–13, 15, 67). Beck et al. (67) evidenced that a Music and Imagery intervention can support improvements in adult displaced people with large effect sizes on PTSD symptoms, sleep quality, well-being, and social functioning. Supporting these findings, Bernard and Dvorak’s (10) systematic review highlights that music-based interventions among displaced populations are effective in alleviating trauma symptoms, enhance coping strategies, and improve overall well-being and sleep quality. In the context of depression, Choi (11) noted that North Korean adolescent refugees in South Korea exhibited significant reductions in depressive symptoms and hopelessness following MT sessions, compared to those participating in art classes. Moreover, Heynen et al. (14) evaluated the ‘Safe & Sound’ MT intervention delivered at the group and individual level in Dutch schools and found that it reduced negative affect and strengthened displaced children’s sense of belonging and inclusion.

This body of research suggests that MT may be a beneficial intervention for PTSD sufferers for several reasons. For some patients, traditional verbal therapies can feel distressing and intrusive (67). Moreover, verbal approaches cannot always integrate the disorganised emotions and sensations that affect the brain, mind, and body of those impacted by traumatic stress (68). As an alternative, MT employs patterned and repetitive rhythmic activities (69) that can bypass higher-order cognitive processes and directly engage more primitive brain regions (70). Neuroimaging studies support this by showing that interacting with music activates limbic and paralimbic regions of the brain - areas responsible for processing emotion, memory, and reward - which may in turn enhance psychological and physiological health (71). In sum, music draws people into a perceived safe and enjoyable therapeutic space that bridges individuals’ experiences between their old world and their new one (72, 73), does not rely on language proficiency, a clear advantage for clients who face linguistic barriers such as displaced people (67) and is both culturally universal (74–76) and beneficial across the lifespan (77, 78). MT has been proposed as a means for traumatised individuals to reconnect with a healthy sense of self (8, 67).

MT has also been shown to be effective in improving relationships between parents and children in families facing difficulties (79). This effectiveness is partly attributed to the close resemblance between early mother-infant interactions and the playful, musical exchanges that occur between a music therapist and child clients. MT allows mothers and children to re-experience – or, in some cases, experience for the first time – the early mother-infant interactions and safely explore control issues in their relationship. It ignites playfulness in adults, allowing them to experience play in a more ‘child-like way’. When parents enjoy playing, their children can sense this enjoyment, which helps foster an initial bond between them (80, 81). MT and music related activities encourage infant directed singing and parent selected songs and have been shown to have a positive effect on the physiological and developmental outcomes of infants (82, 83). For individuals experiencing forced displacement, music from their homeland – whether lullabies, traditional melodies, or hip-hop lyrics – can serve as an essential “first aid kit” for emotional survival. This familiar music may act as a form of psychological aid, offering a sense of control, comfort and cultural continuity during crisis and war situations (84).

Despite this promise, and the fact that MT is non-pharmacological and cost-effective (i.e., intervention applicable to groups rather than individuals), there is considerable lack of research regarding musical interventions with displaced people (10, 85). This research gap contrasts with evidence showing that displaced populations are generally open to exploring innovative treatment approaches (86) and MT may be particularly well suited to meet this need.

The present study aims to bridge this gap and, to our knowledge, it is the first to investigate the benefits of a MT intervention on caregiver-infant dyads in the context of displacement, while also gathering biomarkers data. The only project involving asylum seeking families with young and pre-school children focused on building trust and community engagement (87) but was limited in its assessment of the MT intervention’s outcomes. This project laid the groundwork for the current feasibility study, in which we addressed this research gap by testing an 8-once a week-MT intervention for Ukrainians refugee caregivers and their young children.

### 1.3 The current study

The current feasibility study aimed to shed light on the mental health, parental, social and physiological benefits of an 8-weeks MT intervention as a preliminary step toward informing a randomized controlled study. Conducting the study on a small scale allowed for conceptual refinement of the intervention, and, more importantly, provided valuable insights into how best to implement it while maintaining methodological rigour and capturing a range of outcomes. In Coombes et al. (88) we report the operational details involved in running the programme; herein we focus on the research component of the project, specifically addressing the following research questions:

1. Does the MT intervention improve mental health symptoms, cognitive flexibility and show parental, and social benefits?
2. Does the MT intervention improve parent-infants parasympathetic regulation?
3. Do caregivers report improvements in their children’s development following the MT intervention?

The study focused on Ukrainian displaced families living in the UK following the Home for Ukraine Sponsorship Scheme opened on March 18, 2022, adopting a convergent mixed methods design (standardized tests, questionnaires, physiological biomarkers and structured interviews). This design enabled a thorough and systematic understanding of the MT intervention’s effects, integrating measures of caregivers, their perception of children’s progress, and the caregiver-child relationship, particularly in terms of parasympathetic co-regulation. Thus, the key variables in the present study focused on parents’ mental health (i.e., PTSD, depression, anxiety and wellbeing), cognitive functioning and various aspects of parenting (parental self-efficacy and bonding). In line with previous research highlighting the positive impact of music and singing on affiliation and sense of belonging (12, 14), we also expected that the MT intervention would help caregivers feel more authentic (89), foster a greater sense of shared reality with British people, and increase their interest in social contact. In addition, we expected improvements in parasympathetic nervous system activity, as assessed through electrocardiogram (ECG) and respiration measures. This system plays a key role in emotional reactivity and regulation, and its activity is commonly indexed via RSA (respiratory sinus arrhythmia; 90–92; see Methods section). Higher RSA levels have been associated with greater vagal tone and the capacity for adaptive emotion regulation, especially in safe or socially supportive environments (93). In the context of caregiving, research has shown that mothers holding their infants close exhibit increased RSA responses when exposed to tonal music, suggesting the presence of co-regulation mechanisms between caregiver and infant (94). This is particularly relevant for trauma-exposed populations, where autonomic dysregulation is common. Trauma-affected refugees, such as Iraqi or North Korean migrants, have shown significantly reduced vagally mediated HRV (including RSA), reflecting impaired emotion regulation and heightened physiological arousal (95–97). A meta-analysis of over 50 studies further confirmed that individuals with PTSD consistently display lower resting RSA, supporting the role of parasympathetic withdrawal in trauma-related psychopathology (98). Despite this strong theoretical rationale, few studies have examined RSA in refugee caregivers and their infants, and none, to date, have explored whether MT can enhance vagal regulation in this population.

To address this gap, the current study measured RSA in Ukrainian caregiver–infant dyads before and after the MT intervention. These physiological data were collected alongside validated questionnaires assessing psychological and parenting outcomes (see Methods section). Finally, to deepen our understanding of parents’ experiences, semi-structured interviews were conducted at the end of the MT intervention, exploring caregivers’ reflections on both their own and their children’s development.

## 2 Methods

### 2.1 Participants

Displaced families were recruited with the support of UnityHub Charity. Eligibility criteria for participation required for caregivers to have been displaced from Ukraine following the Russian invasion, to be residing in London (the UK), and to have at least one child aged over 6 months but no more than 2.5 years old. The advertisement clearly stated that the MT group sessions would be held in person at a central London location in the mornings.^1^ The final data consisted of 20 families from two samples that were collected at two separate time points.

#### 2.1.1 Sample 1

This sample comprised of 10 families and one family withdrew from the MT intervention after the first session due to returning to Ukraine. The remaining nine parents (eight females and one male), aged between 30 to 38 years old (*M*=34.33 years, *SD*=2.74) reported that this was the first time they were displaced. Only one parent indicated that while they had not experienced prior displacement themselves, their family had been displaced in 2013/2014 (when Russia annexed Crimea). Parents had between one and three children and the children who attended the MT sessions (three females and five males) ranged in age from 11 to 30 months old (*M*=20.67 months, *SD*=6.04).

#### 2.1.2 Sample 2

This sample comprised of 13 families, and two families withdrew after one session due to conflicting schedules. The remaining 11 parents (all females) were aged between 26 to 38 years old (*M*=32.82 years, *SD*=3.87). The majority reported that this was the first time they were displaced with only one parent being displaced in 2013/2014. Parents had between one and three children and the children who attended the MT sessions (three females and five males) ranged in age from 10 to 33 months old (*M*=17.45 months, *SD*=7.49).

### 2.2 Ethical considerations

Ethical approval for the study was obtained from a Middlesex University Psychology Research Ethics Committee (24360) and the study followed the American Psychological Association Ethical Principles and Code of Conduct and the principles of the Declaration of Helsinki. Participants were explicitly assured of anonymity, confidentiality, and their right to withdraw at any time without providing a reason. Ensuring participants’ welfare and safety was a primary concern throughout the study. During the sessions, the music therapist and the Ukrainian developmental psychologist (present in all sessions) closely monitored participants’ wellbeing to ensure a safe and supportive environment.

### 2.3 Procedure

Participants first completed an online pre-intervention questionnaire (via Momentive) that included brief information about the study, informed consent, demographics and PCL5. They were then invited to a welcoming session where they met the team (comprising the music therapist, two researchers, two music MSc students, a PhD student in developmental psychology, and a Ukrainian developmental psychologist). During this session they received and retained a detailed information sheet in Ukrainian describing the project, their rights, the safeguarding procedure, information on confidentiality and anonymity, supporting resources (in case of unlikely distress) and researchers’ details and informed consent. At the welcoming session, participants completed the remaining quantitative measures in Ukrainian using paper-and-pencil format. They also completed the Schulte’s test (n.d.) in Ukrainian, beginning with a training phase in which they were asked to find and point to the numbers in ascending order as quickly as possible, followed by the actual test. After the eighth (final) session, participants completed a paper-and-pencil survey containing the post-intervention quantitative measures, followed by the Schulte test (n.d.). Next, participants were emailed and asked to complete an online survey that included PCL5. Given concerns about the burden of assessment, sample 2 was limited to completing only the quantitative questionnaire-based measures. A separate debriefing session provided a debrief on the study and thank families.

### 2.4 The intervention

The MT comprised of eight weekly sessions of in-person group MT, each lasting 45–60 minutes. These sessions were delivered by a MA student on a MT course at a UK University, who had prior experience working with displaced families during a clinical placement or an HCPC registered music therapist. In-person sessions took place at a central London location and the study involved four groups of four or six caregiver-child pairs. Drawing on previous experience with similar populations (87, 100), the second author developed the current protocol (**Table 1**). The protocol was informed by the work of 101; 102, who describe using musical activities in classroom settings to support children’s emotional regulation. These activities are designed to incorporate different musical elements — such as loud, soft, fast, and slow — in a structured and intentional way. The protocol was further tailored to be culturally meaningful and representative of the groups, with caregivers actively invited to contribute songs/lullabies to the co-design process. This collaborative approach aimed to enhance parents’ self-efficacy and parenting skills in a manner aligned with their values and belief systems. We also considered to what extent Western practices of group singing and music-making resonate with Ukrainian cultural traditions, and whether these could help displaced caregivers feel safe and authentic. To avoid triggering material, parents were encouraged to identify culturally meaningful songs from their own early childhood musical experiences. These would by their very nature have a repetitive structure with short phrases and a simple melodic line. As a result, all parents agreed that the Ukrainian song ‘*Bulka*’ was most appropriate and was, thus, included in the protocol as a central feature. It was positioned just before the improvisation section, the session’s highest point of energy, where use of instruments were also encouraged as a means of further emotional expression. Each session began with a *greeting song* in which mothers were encouraged to pronounce their child’s name in its original Ukrainian form, with the group joining in to sing each name (an element highly valued by caregivers in post-interviews; see 103) as a symbol of cultural recognition and social integration, essential to those who are separated from their country and community. The music therapist consistently followed the structure outlined in **Table 1** engaging caregivers in musical activities while responding sensitively to children’s contributions, maintaining curiosity and genuine interest in participants cultural backgrounds and musical responses. Therapist responses were shaped according to these musical offerings which grew from the caregivers’ own early musical experiences. Caregivers’ responses to the music were validated and supported by the therapist as “an intercultural meeting beyond words” (104, p. 85) with music acting as a vehicle toward hope and empowerment (105, 106). A variety of musical instruments were used, from guitar and keyboard to smaller handheld items such as shaker, bells and maracas, which were suitable for small hands. All were sanitised before and after each use.

**Table 1:** Structured music therapy protocol: activities and equipment.

Activity	Equipment	Rationale
Greeting song	Guitar	To welcome the group and personalize the experience with each dyad being greeted individually.
Movement activity	Guitar. Circular Stretchy Band of material held by whole group.	To bring the group together in synchrony through a shared movement activity and increase energy levels.
Rhythmic activity	Guitar and shakers	To get the children used to playing instruments together and making music.
Developing the musical timbres and potential of sound.	Guitar, bells, shakers and small drums/tambourines.	To get the children used to playing instruments together and making music. To let them experience different sounds and make choices.
Song from their native land	Guitar and instruments for children	To bring their own culture into the group. The song *Bulka* was used here.
Improvisation	Guitar and all the instruments	This section represents building musical complexity and the high point of energy and dynamism in the session. The children were encouraged to dance and move if they so wished. The therapist brought the energy and dynamics down to close this section.
Soft Lullaby music and vocalization by therapist.	Guitar	Gentle soft ¾ rhythms to offer relaxation and closeness for the dyads.
Goodbye song	Guitar and therapist singing.	Each child to be named and acknowledged as the session ends.

### 2.5 ECG and respiration data

In this study we add an assessment of physiological self-regulation, which plays a crucial role in understanding mental health processes, particularly under chronic stress or trauma. Specifically, we investigated the parasympathetic nervous system activity, a key indicator of emotional reactivity and regulation (90–92). Parasympathetic functioning is responsible for calming the body after stress and supports processes such as emotional resilience, affect regulation, and social engagement. A widely accepted non-invasive method to study parasympathetic regulation is through Respiratory Sinus Arrhythmia (RSA), a component of heart rate variability (HRV) that captures the rhythmic fluctuation of heart rate across the breathing cycle, typically increasing during inhalation and decreasing during exhalation (107, 108).

RSA was measured by collecting three-minute resting-state ECG and respiration measurements before and after the second (baseline) and seventh (post-intervention) MT sessions to understand whether the activities could lead to short-term changes in the parasympathetic activity. These were chosen to avoid meaningful sessions corresponding to the establishment of the therapeutic relationship (i.e., session one), and the ending of the therapy group (i.e., session eight) (as recommended in 109).

In line with established conventions (93, 94), RSA was interpreted as an index of vagal tone and parasympathetic regulation. Higher RSA values are understood to reflect greater physiological flexibility and capacity for adaptive emotion regulation, whereas lower RSA values are associated with autonomic dysregulation and heightened stress responsivity. Thus, increases in RSA from baseline to post-intervention were taken as evidence of improved parasympathetic functioning and enhanced emotional regulation capacity.

### 2.6 Qualitative data

To explore parents’ experiences of the MT group, qualitative data were gathered through individual semi-structured interviews with all nine parent participants. These interviews were conducted in Ukrainian by a Ukrainian psychologist who also supported the sessions, allowing participants to express themselves freely in their native language. Each interview lasted between 90 and 120 minutes and focused on the personal impact of the sessions on themselves, their children, and their parenting. Open-ended questions encouraged reflection on meaningful musical moments, group dynamics, and impressions of the music therapist. All interviews were held virtually via Zoom, recorded, and conducted in a single sitting.

### 2.7 Measures

#### 2.7.1 Mental health outcomes

PCL-5 (99) is a 20-item self-report measure in symptoms related to post traumatic stress disorder according to the Diagnostic and Statistical Manual of Mental Disorders, 5^th^ Edition criteria (DSM-V). Each item (e.g., “*Feeling jumpy or easily startled*?”) is scored from 0 (*no symptom*) to 4, (*extremely disruptive in the previous month*).

PHQ-9 (110) is used to identify depressive symptoms in primary care and it consists of nine questions that assess each symptom of a major depressive episode according to the DSM-V. Symptom frequency over the past two weeks (e.g., “*Feeling down, depressed or hopeless*”) is rated on a Likert scale from 0 (*none*) to 3 (*almost every day*).

GAD-7 (111) screens for anxiety through seven items assessing symptom frequency over the past two weeks (e.g., “*Feeling nervous, anxious or on edge*”), rated on a Likert scale scored from 0 (*never*) to 3 (*almost every day*).

SWEMWBS (112) is a mental well-being scale suitable for the general population, with all items worded to reflect aspects of positive mental health (e.g., “*I’ve been feeling relaxed*.”). The seven items of this scale measure the frequency of the individuals’ attitudes on a 5-point Likert scale from 1 (*none of the time*) to 5 (*all of the time*) with higher scores reflecting a better mental well-being.

#### 2.7.2 Cognitive functioning

Schulte’s test (113, 114) is a non-verbal, culturally equivalent measure that relies on number recognition (rather than letters, thereby avoiding potential confounds related to the Ukrainian sample’s primary use of the Cyrillic alphabet). The test measures concentration and switching of attention. It was presented in a hard copy as a table of 25 cells arranged in a five-by-five grid, with each cell containing a number from 1 to 25 in random order. Responses were recorded in milliseconds from the start until participants identified the final digit.

#### 2.7.3 Music scales

Music@Home questionnaire (115; M@H) measures the self-reported family home musical environment in the early years. It includes two versions: infant (18 items; e.g., *“I teach my child new songs.”*) and pre-school version (17 items; e.g., “*I sing mostly children’s songs or lullabies to or with my child.*”). Responses range from 1 (*completely disagree*) to 7 (*completely agree*).

We also generated 2 items to capture the family’s active engagement with music since the beginning of the war (e.g., “*I noticed that I sing and play music with my child more often since the beginning of the war*” and “*We sing/play musical instruments as a family when we get together.*”). Items scored on a 7-point agreement disagreement scale.

#### 2.7.4 Parenting scales

The Brief Parental Self-Efficacy Scale (116; BPSES) consists of five items measuring a parent’s belief in their ability to effectively perform the parenting role. Participants rate their agreement with each item (e.g., “*I can make an important difference to my child*.”) on a 5-point Likert scale, from 1 (disagree) to 5 (agree).

Mother-to-Infant Bonding questionnaire (117; MIBQ) investigates disturbances in mothers’ feelings towards their newborn. It comprises nine items describing emotional responses (e.g., “*protective*”), each rated on a 4-point Likert scale from 0 (*very much*) to 3 (*not at all*).

#### 2.7.5 Social scales

Authenticity was assessed with two items adapted from English and John (118), measuring how freely caregivers could express their true core selves within the MT environment (e.g., ‘*Right now, it is easy to express my true attitudes and feelings*’ and ‘*Right now, I can be myself*’). Responses ranged from 1 (*strongly disagree*) to 7 (*strongly agree*).

Shared reality expressed one’s motivation to perceive commonalities between their own culture and the relevant outgroup (or host culture), such as shared views on democratic values, socialisation processes, or work ethics. Thus, we adapted three items from Lutterbach and Beelmann (119) by replacing the original stem “Syrian refugees” with “British people” (e.g., “*British people and I share the same outlook on the world.*”). Additionally, we included one item adapted from Firat and Ataca (120) focusing on parenting practices (“*British are not similar to us in terms of family relations and child-rearing practices.*”). Responses ranged from 1 (*strongly disagree*) to 7 (*strongly agree*), with higher values indicating greater shared reality.

Social contact assessed how much caregivers interacted with three groups: their own group (other Ukrainian displaced people), another minority group (displaced people from another country), and the majority group (British people). For each group, we included items adapted from Stathi et al. (121): (a) two items assessing social contact (e.g., “*How often do you interact with your [refugee friends from Ukraine/refugee friends from other countries/British friends]?*”), rated on a 7-point Likert scale from 1 – *never* to 7 – *a lot*; and (b) two items measuring frequency of contact (e.g., “*I am interested in having [friends that are refugees from my own country/friends that are refugees from other countries/British friends].*” rated on a 7-point Likert scale from 1 – *not at all* to 7 – *very much*.

#### 2.7.6 ECG and respiration data

All recordings were conducted in a quiet private room adjacent to the therapy space. Caregivers were seated upright in an armless chair; infants sat on the caregiver’s lap. Breathing was spontaneous (i.e., no paced breathing). The three-minute resting-state ECG and respiration measurements were obtained using the wearable biomedical device BioRadio™ system (Cleveland Medical Devices, Inc., OH). A single BioRadio, standard single-channel ECG registration (II-derivation) was used. For both parents and children, the positive electrode was placed on the lower left side of the chest while the negative one was on the upper right side. A grounding electrode was placed on the upper left side of the chest. The ECG signals were recorded with a 250Hz sampling frequency filtered by a lowpass Bessel filter order 4, with a lower cut-off of 100Hz. The breathing frequency was registered using two thoracoabdominal respiratory effort belts, one placed on the chest and one around the abdominal area. The respiration signal was filtered using lowpass Bessel filter order 2 with a lower cut-off at 1Hz. During each 3-min segment, speaking and singing were not permitted, and participants were asked to remain as still as possible. If an infant became unsettled, the examiner flagged the period using the BioRadio event marker so the corresponding time window could be excluded from analysis.

#### 2.7.7 Semi-structured interviews

This semi-structured interview schedule explored displaced parents’ experiences of participating in a two-month MT group in London with their young children. The first questions unravelled the emotional and contextual backdrop of the participants’ experiences, inviting them to reflect on the impact of displacement and the significance of attending the MT group during a time of crisis. Subsequent questions explored how parents experienced the sessions, including meaningful or memorable musical moments and the dual roles of music-making and therapeutic support. The interviews then focused on the parent-child dynamic, asking how it felt to participate together, how the children responded to the sessions, and whether any changes were observed at home. Later questions addressed perceived personal or parental benefits, shifts in emotional states over the course of the intervention, and whether expectations were met or surpassed. Participants were also asked about any challenges, disappointments, or suggestions for improvement. Finally, the interviews examined group dynamics, impressions of the facilitator, and prior or concurrent engagement in community music activities, closing with space for any additional reflections.

### 2.8 Data analysis

Quantitative analyses were conducted in Statistical Package for the Social Sciences (SPSS, version 28). All mental health and parenting scales were treated as continuous measures by summing responses across all items for each individual scale. For the music scales, we created an overall measure of the M@H questionnaire by averaging all item responses for each individual version (infant vs. preschool) separately; scores were then z-transformed to account for the different number of items used in the two versions. We also created separate mean scores for the two items measuring family active engagement with music, the two items assessing authenticity, and the four items evaluating shared reality. For the social contact measures, we calculated composite social contact scales by averaging the two items for each group (i.e., British, other refugees and Ukrainian refugees). Similarly, for the two items measuring frequency of social contact, we calculated separate average scores for each group. Next, we analysed data as per the general guidelines, by testing for the normality assumption (i.e., Shapiro-Wilk test), which is particularly relevant for small sample sizes (**Table 2**). We thus, computed the differences between the baseline and post-MT intervention measurements and given the normal distribution of all data, paired sample *t*-tests were carried out and effect size calculations were also considered. To note that for the GAD-7, PHQ-9, PCL-5, Schulte’s test and MIBQ, higher scores reflect greater symptom severity or difficulties in the respective constructs, whereas for all other measures, higher scores indicated more favourable outcomes.

**Table 2:** A summary of statistical analyses of mental health outcomes, executing functioning, music scales, parenting scales, and social measures.

Measures	Normality test	Baseline *M* (*SD*)	Follow-up *M* (*SD*)	*M* difference (95% CI)	Effect size
PCL-5	*p = .*111	27.75 (15.94)	18.55 (12.01)	9.2 (3.26, 15.14)	.725
PHQ-9	*p* =.393	8.88 (4.87)	3.23 (3.11)	5.65 (2.86, 8.43)	1.04
GAD-7	*p*=.841	8.5 (5.23)	3.9 (3.00)	4.6 (2.32, 6.88)	.944
SWEMWBS	*p*=.08	24.4 (5.27)	25.25(4.96)	-.85 (-2.62, 0.92)	-.225
Schulte’s test	*p = .*632	111.08 (24.69)	84.51 (27.28)	26.56 (6.27, 46.85)	1.10
M@H	*p*=.278	.001(1)	.01 (1.03)	-.01 (-0.51, 0.48)	-.012
Music after war	*p*=.506	3.39 (1.84)	3.89 (1.52)	-.5 (-1.44, 0.44)	-.256
MIBQ	*p*=.231	4.7 (3.15)	3.8 (2.86)	.9 (-0.63, 2.43)	.276
BPSES	*p*=.436	21.20 (3.02)	21.10 (2.49)	.1 (-0.98, 1.18)	.043
Authenticity	*p*=.124	5.15 (1.65)	5.4 (1.36)	-.25 (-1.03, 0.53)	-.150
Shared Reality	*p*=.357	3.89 (1.39)	4.01 (1.08)	-.13 (-0.67, 0.42)	-.108
Social contact with British people	*p*=.221	3.33 (.78)	3.03 (1.07)	.3 (-0.26, 0.86)	-.251
Social contact with other refugees	*p*=.401	2.48 (1.11)	2.15 (.88)	.33 (-0.14, 0.79)	-.328
Social contact with Ukraine refugees	*p*=.922	2.7 (1.21)	3.28 (1.01)	-.58 (1.38, 0.23)	-.333
Frequency of contact with British people	*p*=.76	.002 (.83)	-0.04 (.73)	.04 (-0.45, 0.53)	.041
Frequency of contact with other refugees	*p*=.747	-.05 (.73)	-.05 (.88)	.002 (-0.43, 0.43)	.002
Frequency of contact with Ukrainian refugees	*p*=.834	.002 (.83)	.001 (.72)	.002 (-0.50, 0.51)	.041

*n*=20 participants (except for the Schulte’s test, which had a *n*=9), *M*, mean; *SD*, standard deviation; *CI*, confidence interval; Effect size, Cohen’s *d*.

RSA values were examined using VivoSense software (Vivonoetics, San Diego, USA). All ECG and respiration signals were visually reviewed to identify and correct artifacts or detection errors. When ectopic beats or misdetections occurred, manual corrections were applied by removing the erroneous segments and performing cubic spline interpolation; corrections were kept below 1%. The timing of R-wave detection was used to derive the time between two consecutive R-waves on an ECG, the RR intervals (RRI). Measures including respiration rate (fR), RRI, and RSA were computed for each participant. To obtain a reliable estimate of RSA during rest, the peak-valley method (94) was applied to three minutes of continuous resting-state data. In order to account for the influence of respiratory frequency on RSA, a within-subject regression analysis was performed, treating RSA as the dependent variable and fR as the predictor (122). Given the considerable inter-individual variability typically observed in RSA measures, within-subject z-transformations were applied to normalise the data (94, 123, 124). To measure the magnitude of the MT effect on RSA values over time, pre-session RSA values have been subtracted from post-session ones for T0 (-pre intervention values) and T1 (-post intervention values) respectively. All data were tested on normality (Kolmogorov–Smirnoff test), homogeneity and sphericity. The final RSA z-values were analysed using a mixed-design ANOVA with *Time* (baseline vs. post-intervention) as a within-subjects factor and *Group* (Parents-RSA vs. Child-RSA) as a between-subjects factor.

For the qualitative data, three researchers (two music therapists and a social psychologist) independently conducted thematic analysis following Braun and Clarke’s (125) framework. Transcripts were transcribed, translated, coded, and thematically organized through collaborative discussion, ensuring analytical rigor and triangulation across disciplinary perspectives.

## 3 Results

### 3.1 Mental health characteristics at baseline

**Table 3** displays participants’ baseline characteristics. Some were single parents displaced in the UK (33%, *n*=7) with a high prevalence of mental health issues: 45% (n = 9) experienced previous trauma and loss-related PTSD (scores ≥ 33, the cutoff indicating probable PTSD; 126), 35% (n = 7) reported anxiety (scores ≥ 10, the validated cutoff in non-clinical settings; 127), and 40% (n = 8) reported depressive symptoms (scores ≥ 10, established cutoff in non-clinical settings; 128).

**Table 3:** Participants’ mental health characteristics at baseline.

Mental Health Measures	Mean (*SD*, range)	*n* (%)
PCL-5		
Severe trauma (≥ 33)	27.75 (16.17, 0-55)	9 (45%)
PHQ-9	8.05 (4.98, 0-17)	
Mild depression (5-9)		6 (30%)
Moderate (10-14)		6 (30%)
Moderately severe (15-19)		2 (10%)
Severe (>20)		0 (0%)
GAD-7	8.5 (5.23, 2-20)	
Mild anxiety (5-9)		7 (35%)
Moderate (10-14)		4 (20%)
Severe (>15)		3 (15%)

### 3.2 Quantitative findings

Post-traumatic stress disorder, as measured by PCL-5, decreased significantly from a baseline mean of 17.75 (*SD*=15.94) to 18.55 (*SD*=12.02), *t*(19) = 3.24, *p*=.004, *d*=.725. Depressive symptoms, as indicated by PHQ-9, decreased significantly from a baseline of 8.88 (*SD*=4.87) to 3.24 (*SD*=3.11), *t*(16) = 4.3, *p*=.001, *d*=1.042. Anxiety levels, as assessed by GAD-7, decreased significantly from a baseline of 8.5 (*SD*=5.23) to 3.9 (*SD*=3.01) following the MT intervention, *t*(19) = 4.22, *p*=.001, *d*=0.944. Mental well-being, as indicated by SWEMWBS scores did not reach statistical significance, *t*(19) = -1.007, *p*=.326, *d* = - 0.225.

Although the current sample was not formally diagnosed we thought is important to examine whether the amount of change these caregivers experienced following the MT reached the threshold for clinical significance, as defined by the minimal clinically important difference (MCID). For post-traumatic stress disorder assessed by PCL-5 the mean change difference of 9.2 (95% CI 3.26, 15.14) met the MCID of 5-10 (129). Depression symptoms measured by PHQ-9 reduced after the MT by 5.65 (95% CI 2.86, 8.43) surpassing the MCID of -1.7 points for clinically meaningful improvement (130). This reduction in MCID is also mirrored for anxiety as measured by GAD-7 as participants exhibited improvements exceeding the MCID of 1.5, with a mean decrease of 4.6 (95% CI 2.32, 6.88) (130).

Attention control and flexibility, as measured by Schulte’s test, improved significantly after the MT intervention from a mean of 111.08ms (*SD* =24.69) to 84.51ms (*SD*=27.28), *t*(7) = 3.10, *p*=.009, *d*=1.10.

However, the MT intervention did not significantly impact M@H exposure (*t*(18) = -.05, *p*=.959, *d*=-.012) or the extent to which Ukrainian families engaged with music since the beginning of the war (*t*(18) = -1.12, *p*=.279, *d*=-.256).

The levels of parental self-efficacy and bonding were not significantly impacted by the MT intervention: BPSES: *t*(19) = .19, *p*=.849, *d*=.043 and MIBQ: *t*(19) = 1.24, *p*=.232, *d*=.276.

Also, there were no significant effects of MT intervention on authenticity (*t*(19) = -.67, *p*=.51, *d*=-.15) and shared reality either (*t*(19) = -.48, *p*=.635, *d*=-.108). Finally, there was no significant impact of the MT intervention on caregivers’ interest to have contact with other Ukrainian displaced people (*t*(19) = -1.49, *p*=.153, *d*=-.333), displaced people from other countries (*t*(19) = 1.47, *p*=.159, *d*=.328) or British people (*t*(19) = 1.12, *p*=.276, *d*=.251). Similarly, following the MT intervention, caregivers did not show an improvement in frequency of social contact with other Ukrainian displaced people (*t*(18) =.18, *p*=.861, *d*=.041) displaced people from other countries (*t*(18) = .009, *p*=.993, *d*=.002) or British people (*t*(18) = .18, *p*=.861, *d*=.041).

In relation to the RSA results, descriptive statistics at pre-intervention (T0) showed that parents had a mean RSA change score (post minus pre) of 0.17 (*SD*=1.05), while infants had a mean change of –0.08 (*SD*=0.49). At post-intervention (T1), the mean RSA change score was 1.07 (*SD*=1.48) for parents and 0.22 (*SD*=0.57) for infants. No statistically significant differences were found between parents and infants in RSA scores at either T0 (*p*=.576, ηp² = .027) or T1 (*p*=.182, ηp² = .144). Results of the mixed-design ANOVA revealed a significant main effect of *Time* (*F*(1,12) = 5.74; *p*=.034, ηp² = .323) showing an improvement in the RSA magnitude from baseline (*M*=.045; *SE*=.22) to post-intervention (*M*=.65; *SE*=.30) and a non-significant main effect of *Group* (*F*(1,12) = 1.43; *p*=.255; ηp² = .106). The interaction between *Time x Group* was also non-significant (*F* (1,12) = 1.42; *p*=.256, ηp² = .106). We followed Chen et al. 2018 (131) recommendations and indicate the pairwise Bonferroni comparison analyses which showed a significant improvement with a strong effect size for the Parents-RSA group over sessions (-0.901, 95% CI [-1.676 to -.127], *p*=.026, ηp² = 0.349), while the Infants-RSA group was not significant (-.302, 95% CI [-1.077 to.421], *p = .*411, ηp² = 0.057).

### 3.3 Qualitative findings

The qualitative analysis yielded three themes concerning mothers’ perceptions of their children’s developmental growth during and after the MT intervention. These themes illuminate how musical engagement supported children’s communication, social-emotional functioning, and the transfer of therapeutic benefits into the home.

#### 3.3.1 Music as a catalyst for communication and language development

Several parents linked their children’s emerging verbal skills to their engagement in musical activities. For children who were previously non-verbal or minimally verbal, participation in musical rituals and singing appeared to stimulate speech. As Inna (pseudonyms are used throughout) remarked, *“When we started the project, he did not* sp*eak. And now he* sp*eaks 2–3 words … maybe music therapy has* sp*ed up the language.”* The musical format provided a motivating and emotionally safe structure that supported vocal expression and communicative attempts, as emphasized by Stefania: *“And during MT, I saw that maybe due to the fact that everything is happening all the time, it is safe, that everyone enveloped you in love the instructor, the professor who came, all this had an effect of calming down. It was nice that they paid attention to me and to my feelings.”*

#### 3.3.2 Growing social engagement and emotional responsiveness

Parents observed significant improvements in their children’s social behaviour, including increased empathy, cooperation, and ability to play collaboratively. These shifts were both witnessed within the sessions and noticed at home. Olivia mentioned that her daughter *“started sharing toys with pleasure. And she liked it. This was not the case before. Because she usually didn’t share toys. And now she began to share with other children on the street.”* Mothers saw these changes as signs of healing and reconnection, often describing their children as “opening up” emotionally. However, the sense of building connections as a group was not confined to the children alone, as described by Oksana: *“As for the music therapy, it was a pleasant time* sp*ent together. It was a nice moment that we could get to know each other, see each other, communicate … and we continue communication with some mothers now.”*

The therapist noted a rise in “mutual responsiveness and gentle interaction” among children who initially had difficulties with emotional regulation: “*I was constantly informing and encouraging, when, towards the end of therapy the anticipation of, “oh, we’ve got this!”…and the children would start getting the toys independently. There was a sense of authorship and leadership … there was a lot more kind of musical communication between the dyads towards the end than there was at the beginning. You know, the circle kind of, it felt together!”*.

#### 3.3.3 Integration of music into daily life as a bridge for continued growth

Musical tools introduced during therapy: songs, instruments, and rituals, were often adopted by parents at home. This continuity appeared to reinforce developmental gains and deepen the parent-child bond. Oksana described how she learned to sing instead of giving commands: *“Not to give commands, but to sing. For example, you can say ‘go to bed’ or you can sing it.”* This theme highlights the important role of music as an enabler of playful communication within families, supporting the continuation of therapeutic progress beyond formal sessions, as illustrated by Marina:*”I enjoyed live music. Live music has a very good effect on a person. Now I’m talking about it and tears are coming to my eyes. Because it was very touching … And live music was very important.”*

### 3.4 The relationship between quantitative and qualitative findings

In **Table 4** we align the quantitative and qualitative findings to provide a more comprehensive overview of the results. Consistent with the convergent mixed-methods design, quantitative and qualitative data were analysed separately but integrated during the final stage of reporting and interpretation to generate insights that either approach might not reveal on its own. This positions the study within an explanatory unidirectional framework for convergent design integration (132). Quantitative findings are complemented by corresponding qualitative themes, which enrich understanding and add depth. The qualitative data gives voice to caregivers’ experiences, offering perspectives on children’s developmental progress and a more holistic view of MT’s effects on both caregivers and children. For instance, although BPSES scores did not reach the threshold for statistical significance, caregivers reported that music became embedded in family routines and may have indirectly supported their parenting practices.

**Table 4:** Relationship between the quantitative and qualitative findings.

Quantitative	Qualitative	Description
Statistically significant improvement in post-traumatic stress disorder, depressive symptoms, and anxiety levels but not wellbeing (PCL-5: *p*=.004; PHQ-9/GAD-7: *p*=.001; SWEMWBS: *p*=.326).	Music as a Catalyst for Communication and Language Development	Caregivers reported that participation in MT sessions helped stimulate speech and communicative attempts in children who were previously non-verbal or minimally verbal.
Statistically significant improvement in cognitive flexibility (Schulte’s test: *p*=.009).	Growing Social Engagement and Emotional Responsiveness	Caregivers and the therapist observed that the MT sessions may have positively influenced children’s social behaviour, including empathy, cooperation, and ability to play collaboratively.
No statistically significant effects on music exposure (M@H: *p*=.959; music engagement after war: *p*=.279)	Integration of Music into Daily Life as a Bridge for Continued Growth	Caregivers noted that musical tools introduced during therapy (i.e., songs, instruments, and rituals) were often incorporated at home, facilitating the continuation of therapeutic progress beyond formal sessions.
No statistically significant effects on parenting scales (BPSES: *p*=.849; MIBQ: *p*=.232).		
No statistically significant effects on social measures (authenticity: *p*=.51; shared reality: *p*=.635; social contact: all *p*s >.153; and frequency of social contact: all *p*s >.861).		
Statistically significant improvement in parent-infant regulation (main effect of *Time*: *p*=.034)		

## 4 Discussion

The findings of the current feasibility study provide initial evidence that MT is an acceptable, affordable, and effective intervention for displaced families experiencing mental health challenges. The *first research question* shows promising results, with statistically significant improvements in PTSD, depression, and anxiety in displaced caregivers. These findings align with prior work indicating a beneficial effect of MT on decreasing the levels of PTSD, both in patients (6, 133–135) and among displaced adults (105). A recent meta-analysis of randomised controlled trials further confirmed that MT has a large therapeutic effect on PTSD, comparable to that of standard psychotherapy (8). Evidence also shows that MT improves anxiety and depression across different clinical contexts (136, 137), including among expectant mothers (138–140) those undergoing caesarean sections (141) and mothers with preterm infants in NICU settings (137). Crucially, given the links between parental mental health and child development, this feasibility study is the first to highlight the broad spectrum of mental health benefits of MT for displaced caregivers, particularly in mitigating the impacts of trauma. Although this migrant sample had no formal diagnosis, the magnitude of change observed in caregivers following the MT was clinically significant (i.e., meeting or exceeding the MCID).

The literature suggests that specific elements of music-based strategies can promote social interaction and engagement in mental health outcomes (10, 142, 143): (1) the group aspect of MT helps people with PTSD in overcoming avoidant behaviour, and (2) musical improvisation, by requiring rapid responses within the music and to the cues and musical expressions offered by others, emphasises active participation (6, 144). Additionally, the current music protocol emphasises repetition, rhythmically stable songs, synchronised physical movement and gradual progression in music-making and improvisation. This design enhances safety, predictability and a sense of control through clear structure and boundaries for expression and experimentation, helping thus to calm the brain heightened stress response triggered by chronic trauma (145). Such musical components have also been shown to reduce anxiety and foster relaxation (146). Along similar lines van Essen et al. (144) argue that for individuals with depression, musical elements, improvisation, and music listening, support emotion regulation through emotional expression. Moreover, music’s intrinsic enjoyment through rhythm, melody and familiarity can stimulate movement, singing and uplift mood.

As part of *the first question*, we also found evidence for improved attentional control and flexibility, potentially emerging as a byproduct of the active music-making. Music naturally attracts attention, helping to divert focus away from invasive or ruminative thoughts (147, 148), a mechanism particularly relevant for traumatized displaced individuals who often experience flashbacks. van Essen et al. (144) suggest that co-creation, verbal reflection, and music listening further enhance attentional control. Additionally, the participatory nature of the MT, particularly during the improvisation, requires heightened awareness and responsiveness, effectively strengthening attention through controlled auditory and behavioural cues. This aligns with de Witte et al.’s (143) framework, which conceptualises attentional engagement and regulation as key mechanisms of change within creative arts therapies. From a neuroscientific perspective, music has been shown to stimulate hippocampal activity and reduce stress, supporting the restoration of motivation, emotional stability, and cognitive flexibility (149, 150).

Results, however, also indicated that part of the quantitative measures did not reach statistical significance, although small descriptive improvements were observed. Beyond the small sample size noted in the limitations, we also acknowledge certain methodological constraints. For example, the M@H measure of the family musical environment showed no significant change following the MT intervention, despite the qualitative findings indicating that music became embedded in family routines (see theme 3). This may be because its subscales focus on parental beliefs, breadth of musical exposure and interaction with instruments rather than broader aspects of music engagement relevant to the intervention. Future studies could use measures assessing whether caregivers feel more empowered and confident using singing or music, to support daily routines (i.e., brushing teeth, washing hands, etc.) following the MT intervention. Results also indicated no impact of the MT intervention on the BPSES or the MIBQ, despite previous research with new mothers suggesting that singing can enhance bonding (151–154). This discrepancy may reflect methodological differences, and it is possible that measures capturing a more state-level emotional closeness between mother and infant would be better suited to detecting the potential benefit of MT, rather than measures of longitudinal bonding.

Qualitative findings from the current research, reported elsewhere (103), indicate that caregivers experienced a sense of comfort, safety, and enhanced group cohesion within the space created by the MT intervention. However, these subjective experiences were not substantiated by quantitative data, which did not reveal significant effects on participants’ sense of authenticity, shared reality, or social contact. Shared reality and social contact measures might be more sensitive to capturing differences in a mixed-cultural group MT intervention rather than a homogenous group (i.e., a solely Ukrainian one). Although a distal aim of the current research was to enhance integration and strengthen connections between community members, the caregivers’ only experience with members of the host society was through their music therapist (in the context of the current intervention). While this was a positive experience (103), it may have been challenging to extrapolate from a single relationship and build up a shared reality that integrated the cultural aspects, traditions, and values of both groups. This is in line with Weston and Lenette (155) findings that in accidental communities, where people are brought together by shared circumstance (in their case an asylum seeker detention centre), musical performance can serve as an expression of collective experience, rather than a form of cross-cultural sharing. Drawing on caregivers’ positive perceptions of friendships formed within their therapy group (as cited in 103) and considering the lack of social support inherent to the condition of a displaced individual, MT is uniquely placed to create an environment where caregivers can develop relationships both within their own culture and across other cultures. These are important steps for people transitioning to new cultures (156–158) and it is possible that over time and within mixed-cultural groups, caregivers may feel increasingly empowered to seek social contact and expand their social networks.

Our *second research question* reveals a clear link between parasympathetic nervous system activity and MT’s delicate processes. The significant increase in participants’ RSA values over time suggests the MT intervention may have supported relaxation and better emotional regulation through enhanced autonomic balance. This is particularly relevant for displaced caregiver–infant dyads, who often experience chronic stress and dysregulation. Arnon et al. (159) and Standley (160) have documented the calming effects of music on preterm infants, and as an extension our MT sessions may have similarly contributed to the observed decrease in stress level over time consequently impacting the RSA values. Van Puyvelde et al. (94) further suggest that music with qualities reminiscent of mother-infant vocal interactions can elicit positive physiological responses in both mothers and infants, fostering co-regulation. Given the nature of our MT sessions, which aimed to replicate these intimate vocal interactions between caregivers and children, it is plausible that the increased RSA levels observed among parents holding their infants close during the sessions reflect enhanced dyadic co-regulation. Elevated RSA is also linked to favourable behavioural reactivity, suggesting a correlation between appropriate vagal regulation and behaviour and emotion regulation (161). In improvisational MT, synchronising musical elements with another person’s play (e.g., beating the rhythm while another person is singing) can support emotional attunement and self-regulation. Evidence from adults with depression suggests that such synchrony fosters emotion regulation and symptom reduction (136, 142), while research on group singing shows it enhances feelings of connection, and belonging (154, 162), indicating parallels with caregiver–infant musical exchanges. In our sessions, synchrony likely emerged through activities such as listening to pleasant music (163, 164), singing pleasant play-songs and lullabies (165), and playing instruments. Each activity provided opportunities for *affect matching*, where the music’s emotional tone aligns with the listener’s affective state, reinforcing emotional processing and bonding (166). These insights advocate for the inclusion of MT within trauma-informed and family-centred care models, especially in contexts of displacement and early adversity.

The qualitative findings address *the third research question* offering valuable insights into the caregivers’ perspectives on the perceived therapeutic outcomes of the MT for their children. First, music acted as a catalyst for communication and language development. Parents reported that their children began vocalizing, singing, or using new words, sometimes for the first time, during sessions, with these expressions often continuing at home. This aligns with research suggesting that predictable, emotionally engaging musical structures can support verbal emergence by scaffolding cognitive and affective processes (102, 167, 168). Second, children demonstrated increased social engagement and emotional responsiveness. Parents observed their children becoming more open, playful, and emotionally attuned within the group and in daily life, consistent with the increases in RSA values. Mothers often described music as a means to regulate both their own and their children, highlighting convergence between biological and experiential indicators of improved co-regulation. These findings echo Bensimon’s (169) trauma-informed model of MT, which highlights the regulatory and relational power of rhythm, repetition, and shared musical time. As in Carr et al. (6), the music group created a safe container for emotional expression, co-regulation, and trust-building. Third, music became integrated into family routines, with parents using songs from the sessions at home for soothing, play, or connection. This ongoing use of music outside therapy served as a bridge for continued growth and relational bonding, reinforcing the role of music as a co-regulatory and empowering medium (170). Together, these themes underscore how culturally sensitive, improvisational MT can foster developmental change, emotional healing, and relational resilience for families navigating trauma and displacement.

Our study addresses Rodwin et al.’s (66) call to explore mediators in MT (that unlike mechanisms, do not fully explain how treatment produces change; 143, 171) by suggesting that MT may facilitate bi-directional regulation in caregiver–infant dyads. For music therapists in clinical practice, gaining deeper insight into these mediators (implicit vs. explicit; common vs. specific) is essential for developing more targeted and efficient treatments, potentially leading to shorter recovery times for displaced caregivers. Our main objective is however, to produce recommendations for a viable intervention scalable to a larger and other refugee population with the aim of identifying key contributors and improve mental health, cognitive functioning and parenting. These recommendations would ultimately be used in a fully developed randomized controlled trial testing the transformational impact of MT in contexts of displacement and integration in different countries, with culturally informed adaptations.

### 4.1 Limitation and future direction

Firstly, as the study has a small sample size and no control group, generalisability is limited as the observed changes could partly be due to regression toward the mean. Addressing these methodological limitations is, therefore, an important consideration for future research in this area. Furthermore, in an increasingly multicultural society, it is important that research actively includes diverse populations, engaging displaced caregivers from varied socioeconomic and cultural backgrounds. Secondly, follow ups are essential to understanding *whether* and *how long* effects are sustained in the long term. Thirdly, the current project raises the possibility that synchronisation between mother and baby following the data from their physiological responses could be contributing factors that explain the positive mental health benefits of the MT. However, more robust research designs should focus on testing the underlying explicit and implicit mediators that promote the mental health benefits of MT. Further clarification is also needed regarding the contribution of factors specific to music-based strategies, as distinct from nonspecific elements (e.g., therapeutic alliance, social bonding, group cohesion) that are common to all group psychosocial interventions. Finally, prioritizing data collection methods that blind both participants and researchers would help reduce the risk of bias related to awareness of outcomes and conditions.

## 5 Conclusion

This mixed methods feasibility study offers preliminary evidence that MT may serve as an effective, non-pharmacological intervention to support the mental health of displaced caregiver–infant dyads. Quantitative findings showed clinically meaningful reductions in PTSD, depression, and anxiety symptoms, improvements in cognitive functioning, and increased RSA levels, indicating enhanced parasympathetic regulation. These results suggest that MT may positively influence both psychological and physiological markers of stress, supporting emotion regulation in trauma-exposed families. Qualitative insights reinforced these outcomes, highlighting perceived developmental gains in children and the integration of music into daily routines as a medium for ongoing co-regulation and bonding. Together, these findings align with emerging evidence on the role of MT in modulating stress-related neuroendocrine pathways and enhancing interpersonal synchrony. While further research involving larger, more diverse samples, and potentially longer interventions (beyond the 8-week format used in this study) is needed to extend these findings, this feasibility study provides a strong foundation for a future randomised controlled trial to evaluate the transformative potential of MT in refugee mental health care.

## Data Availability

The raw data supporting the conclusions of this article will be made available by the authors, without undue reservation.

## Footnotes

1
^1.^ For ethical reasons, all eligible participants were invited despite varying trauma levels, given limited healthcare access. PTSD levels were assessed using the PCL-5 (99), and groups were balanced for moderate and severe symptoms. A Ukrainian Research Fellow, a native speaker with child development expertise, was present during sessions to provide support if needed.
